# Improved CenterNet-Based Multimodal Object Detection for Low-Light and Complex Environments

**DOI:** 10.3390/s26123735

**Published:** 2026-06-11

**Authors:** Zhigang Yao, Hengxin Xu, Huazhong Zhang, Xiaoguang Tu, Juhang Yin

**Affiliations:** 1 College of Aviation and Electronics and Electrical, Civil Aviation Flight University of China, Guanghan 618307, China; xyhk_yzg@163.com (Z.Y.); xhx13133151627@163.com (H.X.); txg198955@163.com (X.T.); yinjh0121@163.com (J.Y.); 2 School of Communication and Information Engineering, Chongqing University of Posts and Telecommunications, Chongqing 400065, China

**Keywords:** object detection, low-light conditions, complex environments, CenterNet

## Abstract

To address insufficient detail representation, inadequate cross-modal fusion, and limited localization accuracy in object detection under low-light and complex background conditions, this study proposes an improved CenterNet-based multimodal object detection method. The model uses fused images and infrared images as dual-source inputs, where infrared wavelet priors are introduced to enhance texture and structural representation. A Feature Fusion Attention (FFA) module is designed to improve cross-modal feature interaction, while a Heatmap-Guided Detection Head (HGDH) is introduced to explicitly enhance target-related regions during detection. In addition, a two-stack Hourglass backbone is adopted for multi-scale modeling of global semantics and local details. Based on the public LLVIP dataset, a precisely paired fused-image/infrared-image dataset, termed RH-25, was constructed for experiments. On the RH-25 dataset, the proposed method achieves a 3.51% improvement in mAP@0.5 relative to the baseline, as demonstrated by comparative and ablation experiments. Moreover, supplementary experiments on the MFAD dataset indicate potential cross-dataset adaptability under different scenes and multi-class conditions. These results indicate that the proposed method can improve detection performance in low-light and complex environments.

## 1. Introduction

With the continuous development of object detection technology, autonomous sensing systems have been widely applied in military security, critical area surveillance, and civilian inspection tasks [1]. Therefore, real-time object detection for security threats such as covert reconnaissance and intelligence theft in airports and other highly regulated areas is of great significance. However, in such scenarios, factors including complex backgrounds, low-light conditions, and limited target visibility often lead to degraded detection accuracy and, in severe cases, even make targets difficult to identify, thereby significantly affecting the reliability of detection systems [2,3]. In view of these challenges, the development of robust object detection methods for low-light and complex environments is of considerable theoretical significance and practical value.

Against this background, multimodal object detection based on visible and infrared images has gradually become an active research topic [4,5]. Compared with single-modality approaches, infrared images can provide relatively stable thermal radiation information, whereas visible images or fused images preserve richer texture and edge details. As a result, their combination usually exhibits stronger environmental adaptability under complex scenes and low-light conditions [6,7]. Owing to the swift progress of deep learning, CNNs now serve as a foundational pillar for research in multimodal object detection, due largely to their strong feature extraction ability [8,9]. As reported in previous work, CNN-based multimodal approaches primarily leverage complementary information from visible and infrared images via end-to-end joint training, dual-branch feature extraction, and cross-modal feature fusion, which in turn enhances detection robustness in low-light, occluded, or complex background scenarios [10].

On this basis, some studies have further introduced multimodal fusion strategies into keypoint-based detection frameworks to improve target localization and recognition performance. Among them, multimodal object detection methods based on CenterNet have attracted increasing attention in recent years [11]. To improve object detection under low-light, low-contrast, occluded, and complex-background conditions, current methods typically introduce cross-modal feature interaction, pixel-level fusion, or feature-level enhancement mechanisms into the CenterNet framework. Overall, although current methods have made progress in exploiting multimodal information, their robustness in low-light and complex-background scenarios remains limited [12].

**Figure 1:**
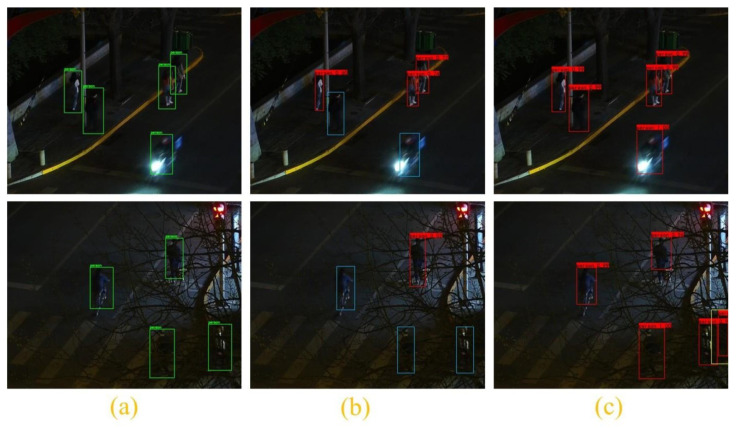
Representative failure cases from existing methods on the RH-25 test set under low-light and complex environments. (**a**) Visible-light image with ground-truth annotations; (**b**) RT-DETR detection results; (**c**) FreDet detection results. Green boxes indicate ground truth, red boxes indicate detections, blue boxes indicate missed detections, and yellow boxes indicate false positives.

Although the aforementioned methods have achieved a certain level of progress in multimodal object detection, as illustrated in Figure 1, representative existing methods such as RT-DETR and FreDet still suffer from typical failure cases, including missed detections and false positives, when targets are weakly illuminated, partially occluded, or surrounded by cluttered backgrounds [13]. First, most existing approaches rely on feature complementarity between different modalities to improve detection accuracy. However, under low-light conditions, the texture and edge details of some targets often degrade severely and may even become difficult to observe effectively in visible images, thereby limiting the performance gains brought by multimodal complementarity. Second, infrared and visible images differ significantly in imaging mechanisms and feature representations, and direct fusion may easily introduce information loss, feature misalignment, and insufficient fusion, which in turn affect detection performance [14]. Third, the detection stage usually lacks explicit guidance for target-related regions. As a result, the localization and discrimination capabilities of the detector remain limited in scenarios involving complex backgrounds, occlusion, and weak responses. These issues indicate that it is still necessary to further investigate how to jointly optimize object detection in low-light and complex environments from the three aspects of input construction, cross-modal fusion, and detection enhancement.

To address the aforementioned challenges, this work presents an enhanced CenterNet-based multimodal object detection method [15]. The proposed framework uses fused images and infrared images as dual-source inputs, introduces infrared wavelet detail priors for structural representation, and incorporates FFA and HGDH modules for cross-modal feature refinement and target-region enhancement [16]. A two-stack Hourglass backbone is adopted for multi-scale feature modeling [17].

The main contributions of this paper can be summarized in the following three aspects:

1. A multimodal object detection framework based on the improved CenterNet is proposed, which incorporates Haar wavelet detail priors from infrared images into the overall network. This framework establishes a dual-source input mechanism driven by the semantic information of the fused images and the texture structure of infrared images, thereby enhancing the input representation capability in low-light and complex scenes.
2. A Feature Fusion Attention (FFA) module is designed to spatially align and achieve early fusion of the semantic features of fused images with the multi-sub-band detail features of infrared wavelets. In addition, a SimAM-based lightweight attention enhancement and a residual stable injection strategy are employed to achieve efficient cross-modal expression of semantic and texture information.
3. A Heatmap-Guided Detection Head (HGDH) module is designed, which explicitly selects target-related features by generating spatial probability masks using the predicted heatmaps. This module, combined with contextual modeling and lightweight attention enhancement mechanisms, improves target localization accuracy and detection robustness in complex scenes.

## 2. Related Work

This section mainly reviews studies on visible and infrared image-based object detection, as well as CenterNet-based detection models closely related to this work adopt dual inputs of fused images and infrared images.

### 2.1. Object Detection with Visible and Infrared Images

Under complex environments and low-light conditions, visible and infrared images exhibit different advantages and limitations. Visible images can provide rich texture and detail information, but their representation capability is often significantly restricted under low illumination and complex background conditions. In contrast, infrared images can stably provide the thermal radiation information of targets, but they usually lack sufficient appearance details. Thus, fusing visible with infrared data for object detection has become a major research direction within complex-scene understanding [18,19]. Deep learning-based detection methods are typically divided into two categories: two-stage and one-stage approaches. The first category typically offers higher detection precision, while the second proves more appropriate for scenarios with strict real-time demands [20,21].

### 2.2. Multimodal Object Detection Based on Fused Images

Recently, multimodal feature fusion of visible and infrared imagery has been widely adopted to address the shortcomings of single-modality data. Existing studies have proposed methods that unify image fusion and object detection into an end-to-end joint optimization framework, enabling the collaborative training of fusion tasks and detection tasks. Other studies have employed feature-based image fusion strategies, which have demonstrated the improvement in object detection accuracy through fused images [22,23]. To address the challenge of insufficiently modeling the complementary information between modalities in fusion results, some research further introduces decision-level fusion methods to enhance detection performance, while other studies use adaptive weighting strategies to improve detection accuracy under low-light and high-noise conditions [24]. On the whole, current fusion-based detectors have achieved substantial gains in detection accuracy. However, under low-light and even nighttime conditions, how to construct more complementary dual-modality representations and enhance cross-modal feature interactions remains a problem worthy of further investigation.

### 2.3. CenterNet Detection Models with Dual-Modal Inputs

As a typical anchor-free detector, CenterNet has been widely applied in object detection tasks owing to its efficient architecture, suitability for multi-scale targets, and compatibility with complex backbone networks. In recent years, studies on improving CenterNet have mainly focused on backbone optimization, the introduction of attention mechanisms, and feature enhancement. Existing research has improved infrared feature extraction by adopting stronger backbones combined with channel attention mechanisms, while denoising preprocessing modules have also been introduced to enhance the quality of infrared inputs. Other studies have incorporated channel–spatial attention mechanisms into the original CenterNet framework and combined them with feature selection modules to achieve cross-level feature aggregation. In addition, some methods have enhanced feature representation capability and receptive field through backbone replacement, dilated convolutions, and lateral connections [25,26]. Although these methods can enhance detection performance to some degree, they still have inherent limitations, most of them are still developed based on single-modal features or only enhance network representation capability through relatively simple attention mechanisms. Under low-light and complex environmental conditions, it remains worthwhile to further investigate how to construct a dual-modal input paradigm jointly driven by fused images and infrared images, while simultaneously alleviating information loss during fusion, strengthening modal complementarity, and improving the discrimination ability of the detection head for target-related regions.

## 3. Materials and Methods

### 3.1. Overall Network Architecture

**Figure 2:**
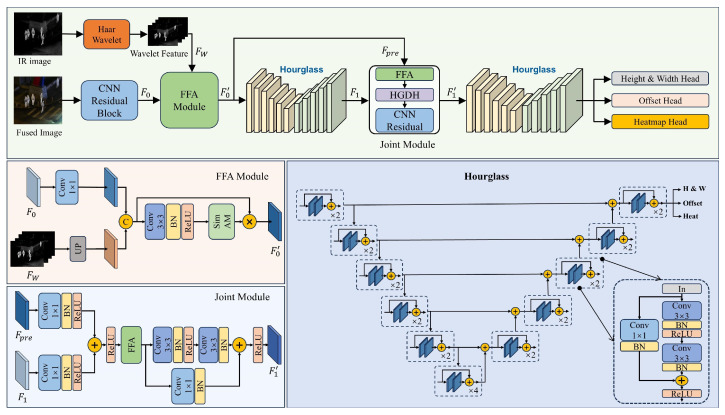
Overall architecture of the proposed multimodal object detection framework. The network takes the fused image and infrared image as dual-source inputs. The joint section indicates the feature aggregation process among the FFA-enhanced features, heatmap-guided responses, and CNN residual features, rather than an independent detection branch. The aggregated features are further processed by the Hourglass backbone and the CenterNet detection head for final prediction.

The proposed method is built upon the CenterNet keypoint-based detection framework and aims to extract and learn target-related features from fused images and infrared images. CenterNet is selected as the base detection framework because its anchor-free keypoint-based detection paradigm is consistent with the design of the proposed method. Its heatmap-based prediction structure naturally supports the HGDH module, which uses heatmap-derived spatial responses to enhance target-related regions. Moreover, the heatmap, object size, and offset prediction branches provide a clear framework for evaluating the proposed FFA and HGDH modules. When combined with the Hourglass backbone, CenterNet can also perform multi-scale feature modeling for low-light and complex-background detection. The overall network consists of a dual-source input guided by wavelet priors, cross-modal feature fusion and enhancement, a two-stack Hourglass backbone, a Joint feature processing module, and a three-branch detection head. An overview of our model’s architecture is presented in Figure 2. In this study, the RH-25 dataset mainly focuses on pedestrian detection under low-light and complex environments, and the proposed model follows a pipeline of “fused-image semantic input → infrared wavelet-prior-guided fusion → stacked Hourglass backbone → CenterNet detection head”. RGB images are not used as an additional independent input branch in this architecture. The fused image is adopted as the semantic input because it preserves visual appearance information from the visible image, while the infrared image is retained to provide thermal responses and wavelet-based structural-detail priors. This two-source design reduces input redundancy and computational cost while maintaining complementary multimodal information.

At the input stage, the infrared image first undergoes two-dimensional Haar wavelet decomposition to extract multi-resolution structural-detail priors. Haar wavelet decomposition is adopted because it can efficiently separate the infrared image into a low-frequency approximation component and horizontal, vertical, and diagonal high-frequency detail components. Compared with simple downsampling or denoising, the proposed operation explicitly preserves the high-frequency sub-bands and introduces them into the subsequent feature fusion stage. These detail components provide boundary, contour, and local intensity-variation cues, which are useful for enhancing target representation under low-light and complex-background conditions [27], which can be expressed as

(1) $$\mathrm{coeffs}=\mathrm{wavedec}2\left(I_{ir}\times 255,\;\mathrm{haar},\;\mathrm{level}=1\right)$$

(2) $$F_{\mathrm{wave}}=\mathrm{Concat}\left(c_{A},c_{H},c_{V},c_{D}\right)\in R^{\frac{H}{2}\times \frac{W}{2}\times 12}$$

where $I_{ir}$ denotes the input infrared image, and $c_{A}$, $c_{H}$, $c_{V}$, and $c_{D}$ represent the approximation sub-band and the horizontal, vertical, and diagonal detail sub-bands, respectively. In the fused-image branch, the input first passes through a convolutional layer and a residual block for downsampling and preliminary semantic feature extraction, producing the fused-image feature $F_{pre}$.

Subsequently, in the Feature Fusion Attention (FFA) module, the fused-image feature and the infrared wavelet feature are first matched to the same spatial resolution at the feature level and then fused along the channel dimension. Through this module, the network can effectively inject infrared texture details while preserving the fused image’s semantic content, thereby improving the quality of cross-modal feature fusion.

In the backbone stage, the fused feature is fed into a two-stack Hourglass network for multi-scale modeling. Through its symmetric downsampling–upsampling structure and cross-layer skip connections, the Hourglass network repeatedly integrates global semantic information and local details. The output of the first Hourglass is fused with the previous-layer feature in the Joint module, where the feature is further enhanced by combining wavelet priors and the heatmap-guided mechanism. Finally, the output of the second Hourglass is sent to a CenterNet-style three-branch detection head, which predicts the center heatmap, object size, and center offset, thereby producing the final detection result [28].

By cascading the above modules, the proposed network achieves collaborative modeling of fused-image semantic information, infrared wavelet texture priors, and target-region spatial responses within an encoder–decoder multi-scale architecture, thus boosting detection accuracy and robustness under poor-illumination and complex-background situations.

### 3.2. Feature Fusion Attention (FFA) Module

**Figure 3:**
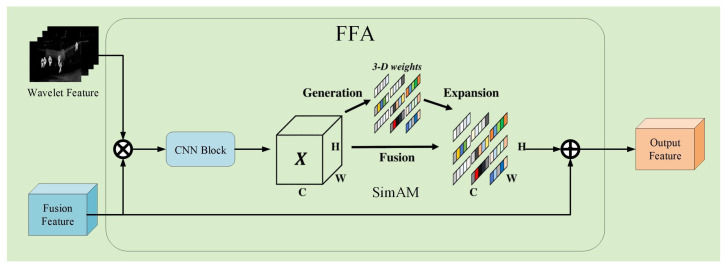
Structure of the FFA module. The joint section denotes the fusion of infrared wavelet structural-detail features and fused-image semantic features. The concatenated features are refined through 1×1 convolution, SimAM-based attention, and residual injection, so that structural-detail cues can be introduced while preserving the original semantic representation.

Compared with conventional multimodal methods that either perform feature concatenation only at high-level stages or rely on parameterized attention mechanisms, the proposed FFA module first spatially aligns and achieves early fusion of infrared wavelet detail features with the semantic features of fused images at the front end, thereby alleviating the loss of texture and edge information in low-light and occluded scenarios [29]. In addition, the module introduces a lightweight SimAM-based attention enhancement mechanism, which can generate three-dimensional adaptive weights without introducing extra channel-wise or spatial convolutions. This design suppresses noise responses and enhances key regions with relatively low parameter and computational overhead. Furthermore, a residual fusion strategy with a learnable scaling coefficient γ is adopted to preserve the stability of the original semantic representation while injecting infrared detail information, thereby reducing feature distortion caused by excessive reliance on attention. Overall, as shown in Figure 3, the FFA module helps alleviate the problems of insufficient detail representation under low-light and noisy conditions, feature redundancy caused by direct concatenation, and instability during the fusion process, while improving cross-modal fusion quality and detection robustness with low computational cost [30].

The proposed Feature Fusion Attention (FFA) module is illustrated in Figure 3. First, the feature $F_{\mathrm{pre}}$ extracted from the fused-image branch and the multi-sub-band feature $F_{\mathrm{wave}}$ obtained from the infrared image through Haar wavelet decomposition are spatially aligned and concatenated along the channel dimension to form a joint representation, which can be expressed as

(3) $$F_{c}=\mathrm{Concat}\left(F_{\mathrm{pre}},\mathrm{Pool}\left(F_{\mathrm{wave}}\right)\right)$$

where $F_{\mathrm{pre}}$ denotes the semantic feature extracted from the fused-image branch after downsampling, and $F_{\mathrm{wave}}$ denotes the multi-sub-band texture feature obtained from the infrared image through Haar wavelet decomposition. We apply Pool(·) to resize the wavelet feature spatially, matching the dimensions of the fused-image feature, and Concat(·) denotes channel-wise concatenation. Here, “spatial alignment” refers to feature-level resolution matching rather than geometric registration or pixel-intensity consistency. Pool(·) is used to resize the infrared wavelet feature to the same spatial size as $F_{\mathrm{pre}}$ for channel-wise concatenation.

Subsequently, the concatenated feature is compressed and linearly fused by a 1×1 convolution to obtain the intermediate feature $F_{f}$:

(4) $$F_{f}={\mathrm{Conv}}_{1\times 1}\left(F_{c}\right)$$

where ${\mathrm{Conv}}_{1\times 1}$ is used to reduce the channel dimension of the concatenated feature and generate the fused intermediate feature $F_{f}$, which jointly captures semantic content from the fused image and textural cues from the infrared image. On this basis, SimAM is employed to perform lightweight attention enhancement on $F_{f}$. For a given neuron $F_{f}\left(i,j,c\right)$, the inverse energy score $e_{t}$ is computed according to intra-channel spatial statistics as follows:

(5) $$e_{t}\left(i,j,c\right)=\frac{{\left(F_{f}\left(i,j,c\right)-\mu_{c}\right)}^{2}}{4\left(\sigma_{c}^{2}+\lambda \right)}+\frac{1}{2}$$

where $\mu_{c}$ and $\sigma_{c}^{2}$ denote the mean and variance of channel *c*, respectively, and λ is a small regularization coefficient.

The weighted feature $F_{\mathrm{att}}$ is then obtained by applying the Sigmoid function to the inverse energy score:

(6) $$F_{\mathrm{att}}\left(i,j,c\right)=\sigma \left(e_{t}\left(i,j,c\right)\right)\cdot F_{f}\left(i,j,c\right)$$

where σ(·) denotes the Sigmoid function, and $F_{\mathrm{att}}$ denotes the adaptively enhanced feature used to highlight target-related structural responses.

Finally, a residual fusion strategy is adopted to combine the enhanced multimodal feature with the original semantic feature from the fused-image branch, so as to preserve semantic stability while injecting infrared texture details. This process can be formulated as

(7) $$F=\gamma \cdot F_{\mathrm{att}}+F_{\mathrm{pre}}$$

where γ is a learnable scaling coefficient used to control the contribution of the attention branch. The final output feature *F* is then fed into the subsequent Hourglass backbone and Joint module, thereby providing a more stable cross-modal feature representation for the downstream detection task.

**Figure 4:**
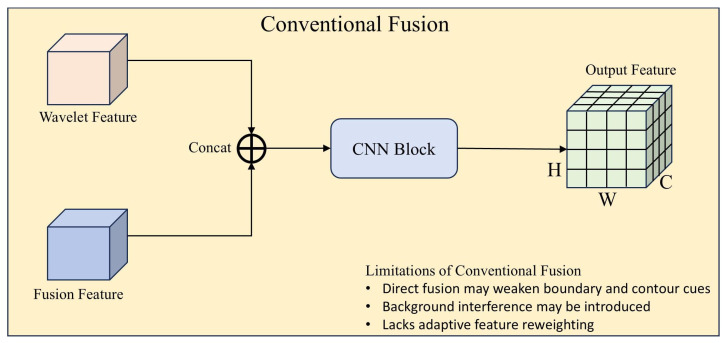
Structure of a conventional fusion strategy and its limitations. The wavelet feature and fused-image feature are directly concatenated and then processed by a CNN block. This direct fusion strategy may weaken boundary and contour cues or introduce background interference, and lacks adaptive feature reweighting.

To further clarify the difference between conventional fusion and the proposed FFA module, a schematic comparison is provided in Figure 4. In conventional fusion, the wavelet feature and the fused-image feature are usually directly concatenated and then processed by a CNN block. Although this strategy is simple, it lacks adaptive feature selection and may weaken boundary and contour cues while introducing background interference. In contrast, the proposed FFA module first matches the infrared wavelet feature to the same spatial resolution as the fused-image feature and then performs channel-wise concatenation, as formulated in Equations (3)–(7). The subsequent 1×1 convolution learns cross-modal channel interactions, while the SimAM-based attention mechanism adaptively enhances informative structural responses and suppresses less relevant background activations. Finally, the residual injection strategy $F=\gamma \cdot F_{\mathrm{att}}+F_{\mathrm{pre}}$ preserves the original semantic representation while introducing infrared structural-detail information. Therefore, FFA alleviates the loss of edge and local structural information under low-light and occluded conditions through structural-detail priors, adaptive attention enhancement, and stable residual fusion.

### 3.3. Heatmap-Guided Detection Head (HGDH)

**Figure 5:**
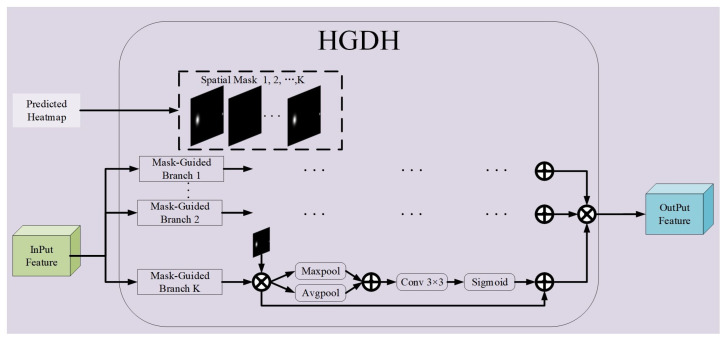
Structure of the Heatmap-Guided Detection Head (HGDH) module, including spatial mask-guided branches, feature aggregation, and context-aware enhancement with pooling and convolutional operations.

Compared with conventional global channel attention or simple feature concatenation strategies, as shown in Figure 5, the proposed Heatmap-Guided Detection Head (HGDH) generates spatial probability masks from the predicted heatmaps to explicitly select target-related features. It further constructs a lightweight attention enhancement mechanism by combining pooling operations, a 3×3 convolution, and Sigmoid activation, and finally improves feature representation through feature aggregation and residual reintegration. This module has the advantages of explicit target guidance as well as relatively low parameter and computational overhead. It can more effectively suppress background noise and enhance target-region responses in scenarios involving weak target responses, dense target distribution, and occlusion, thereby alleviating the limitations of traditional concatenation or global attention mechanisms in target-region localization, such as insufficient sensitivity and redundant feature information [31,32].

The proposed Heatmap-Guided Detection Head (HGDH) is illustrated in Figure 5. The module first generates spatial probability masks from the feature map $F_{\mathrm{in}}$ output by the previous Hourglass stack and the corresponding heatmap $H_{\mathrm{hm}}$, and then explicitly selects target-related features based on these masks. In HGDH, the spatial mask is generated from the intermediate heatmap $H_{\mathrm{hm}}^{\left(1\right)}$ predicted after the first Hourglass stack, rather than from the final detection head. The softmax operation is applied over the spatial dimensions of this intermediate heatmap to obtain *M*. No stop-gradient operation is applied during training, so gradients can be propagated through the intermediate heatmap prediction branch. During inference, the same forward data flow is used without gradient computation. This process can be expressed as

(8) $$M={\mathrm{Softmax}}_{h,w}\left(H_{\mathrm{hm}}^{\left(1\right)}\right)$$

(9) $$F_{m}=F_{\mathrm{in}}⊙M$$

where $F_{\mathrm{in}}$ denotes the input feature map, $H_{\mathrm{hm}}^{\left(1\right)}$ denotes the intermediate heatmap predicted after the first Hourglass stack, and *M* denotes the spatial probability mask obtained by normalizing the heatmap over the spatial dimensions. Through this process, the network can exploit the spatial prior provided by the prediction results to preliminarily enhance target-related regions.

Subsequently, the selected feature is processed by max pooling and average pooling to extract contextual information, and a lightweight 3×3 convolution together with Sigmoid activation is used to generate attention weights:

(10) $$P=\mathrm{MaxPool}\left(F_{m}\right)+\mathrm{AvgPool}\left(F_{m}\right)$$

(11) $$A=\sigma \left({\mathrm{Conv}}_{3\times 3}\left(P\right)\right)$$

where MaxPool(·) and AvgPool(·) are used to extract contextual information. We apply a 3×3 convolution (${\mathrm{Conv}}_{3\times 3}$) to produce the attention weight map, while *A* represents the spatial attention response following Sigmoid activation.

Subsequently, the selected feature is reweighted by the attention weights, while feature aggregation together with a residual connection further improves the stability of the output representation. This process can be written as

(12) $$F_{\mathrm{out}}=F_{m}⊙A$$

where $F_{\mathrm{out}}$ denotes the output feature enhanced by heatmap guidance and attention weighting, which can be further fed into the subsequent decoding detection head, while the residual connection helps maintain training stability. Overall, the HGDH module effectively highlights target-region responses and suppresses background noise through heatmap-guided spatial probability masks, contextual modeling, and lightweight attention enhancement, thereby improving the discriminative capability and stability of the subsequent detection head.

### 3.4. Hourglass Backbone

In this study, a two-stack Hourglass network is adopted as the backbone to achieve multi-scale modeling of global semantics and local details. Through its symmetric downsampling–upsampling structure and cross-layer skip connections, the Hourglass network repeatedly integrates features at different scales during the encoding–decoding process, thereby enhancing the representation capability of the model for multi-scale targets in complex scenes. Its output can be abstractly expressed as

(13) $$Y=U+\mathrm{Up}(D_{3})$$

where *U* denotes the upper-branch feature, and $D_{3}$ denotes the feature encoded by the lower branch through multi-scale processing. The two-stack design is adopted to further improve the stability of target localization and regression under low-light and complex-scene conditions.

### 3.5. Collaborative Mechanism of the Proposed Modules

The proposed method tackles low-light and complex-scene object detection through the collaboration of FFA, HGDH, and the two-stack Hourglass backbone. Specifically, FFA introduces infrared wavelet structural-detail priors into the fused-image semantic feature, thereby improving the preservation of boundary, contour, and local structural cues. The two-stack Hourglass backbone then performs multi-scale feature modeling to integrate global semantics and local details for targets of different scales. Finally, HGDH uses heatmap-derived spatial probability masks to enhance target-related regions and suppress background interference during detection.

These modules are not independent components but form a progressive enhancement pipeline. FFA first improves cross-modal feature representation, the Hourglass backbone further refines the features at multiple scales, and HGDH guides the detection head to focus on target-relevant spatial responses. Their combined interaction improves structural-detail representation, multi-scale modeling, and target-region discrimination, which jointly addresses the challenges of low illumination, occlusion, complex backgrounds, and weak target responses.

## 4. Results

This section first introduces the RH-25 dataset used to evaluate the proposed bimodal object detection model. Next, we outline the setup and implementation specifics of our experiments. Using this foundation, we carry out organized ablation experiments aimed at assessing our modules’ effectiveness. Furthermore, our method is benchmarked against both conventional approaches and representative state-of-the-art detectors to evaluate its performance under the current experimental setting. Additionally, we conducted extra experiments on the MFAD autonomous driving dataset to further assess the cross-dataset adaptability of our method across varying scenarios and multiple target types.

### 4.1. Datasets

To validate the efficacy of our enhanced model, we performed experiments using two datasets acquired from distinct scenarios. These two datasets cover a variety of scenes ranging from daytime to nighttime, and the main detection targets include pedestrians, vehicles, and other objects, with particular emphasis on low-light and complex environmental conditions. Among them, the RH-25 dataset contains 9353 image pairs, while the MFAD dataset contains 12,194 image pairs. Each dataset was divided into training, validation, and test sets at a ratio of 7:2:1. The construction process and screening criteria of the RH-25 dataset developed in this study are described in detail below.

#### 4.1.1. RH-25 Dataset

**Figure 6:**
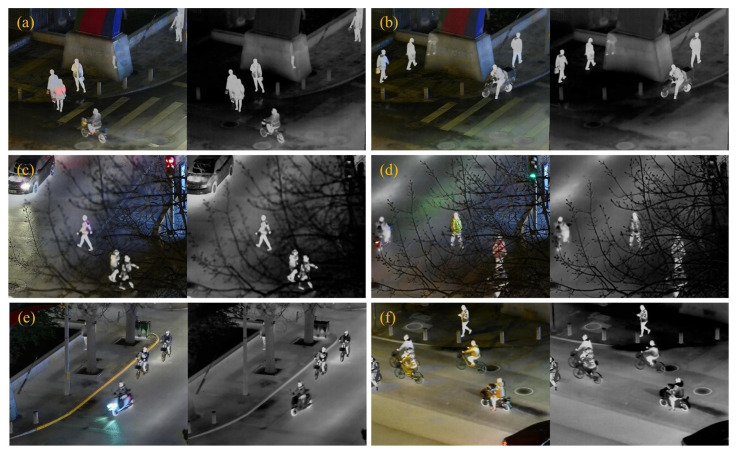
Visualization of RH-25 dataset samples. (**a**,**b**) Fused-image and infrared-image pairs captured under low-light conditions; (**c**,**d**) fused-image and infrared-image pairs with occlusion; (**e**,**f**) fused-image and infrared-image pairs in complex environments.

This dataset was constructed based on the public LLVIP dataset. Images captured under low-light and complex environmental conditions were first selected, and image fusion was then performed to generate a dataset composed of precisely paired fused images and infrared images. The fused images were generated using SeAFusion, a semantic-aware infrared and visible image fusion network proposed by Tang et al. [33]. SeAFusion was used as an offline frozen preprocessor and was not jointly trained with the detector. The pre-generated fused images and the corresponding infrared images were then used as fixed dual-source inputs for detector training and inference. RH-25 mainly contains low-illumination and complex-scene samples captured from a high-altitude top-down perspective, with pedestrians as the primary detection target. Representative examples of the dataset are shown in Figure 6.

#### 4.1.2. Screening Criteria

In previous studies, low-light images are usually screened based on brightness and noise-related statistical features. In general, when an image has relatively low mean brightness and a relatively high noise level, it can be regarded as exhibiting typical low-light characteristics [34]. The corresponding scoring form can be expressed as

(14) S=α·MeanBrightness+β·NoiseStd.Dev

In the low-light screening process, images with a mean brightness lower than 50 were first selected as low-light candidates. The weighting coefficients in Equation (14) were set to α=0.7 and β=0.3, where brightness degradation was considered the primary factor and noise level was used as an auxiliary factor. For complex-environment screening, the edge-density threshold was set to 0.3, and the noise standard deviation threshold was set to 30, using edge density and noise variation as complexity indicators [35]. Images satisfying both the low-light and complex-environment criteria were selected to construct the RH-25 dataset. The corresponding scoring form can be written as

(15) Score=EdgeDensity+LightingVariation

Based on the above criteria, images with a mean brightness lower than 50 were defined in this study as low-light images, and complex-environment samples were further screened by combining the indicators of edge density and lighting variation. Ultimately, images that simultaneously satisfied both the low-light criterion and the complex-environment criterion were selected as the data samples used in this study. In this work, complex environments mainly refer to scenes with complex background textures, rich edge information, obvious local illumination variations, and a certain degree of occlusion interference.

### 4.2. Experimental Setup

#### 4.2.1. Experimental Environment and Parameter Configuration

To guarantee fairness and replicability, we performed all experiments under identical hardware and software conditions. The experimental setup consisted of the following: Windows 11 Professional (Microsoft Corporation, Redmond, WA, USA), an Intel(R) Core(TM) i9-14900K CPU (Intel Corporation, Santa Clara, CA, USA), an NVIDIA RTX A4000 GPU (NVIDIA Corporation, Santa Clara, CA, USA), CUDA 13.0, and PyTorch 2.4.1 as the deep learning framework. To ensure consistency in the comparison experiments, all models adopted the same training strategy. Specifically, the Adam optimizer was used for training, with 200 epochs, a batch size of 8, and an input image size of 512×896 pixels. The initial learning rate and the minimum learning rate were set to $1\times 10^{-5}$ and $1\times 10^{-6}$, respectively. A cosine annealing learning rate schedule was employed, and model checkpoints were saved every 10 epochs. These parameter settings were determined by considering factors such as dataset size, input resolution, and training stability.To ensure fair comparison, all comparison experiments were conducted under the same dataset split, input resolution, training strategy, evaluation thresholds, and hardware environment. The reported results were obtained from a fixed experimental setting, and no per-model threshold tuning was applied.

#### 4.2.2. Evaluation Metrics

To thoroughly evaluate model performance, we adopted a range of metrics: Average Precision (AP), Mean Average Precision (mAP), Precision, Recall, Frames Per Second (FPS), and the F1 score. Specifically, AP and mAP quantify the overall detection capability of the model, Precision and Recall indicate the precision and completeness of detections, respectively, and the F1 score assesses the trade-off between these two metrics. FPS is used to represent the model’s detection speed. The Intersection over Union (IoU) threshold was set to 0.5 for all metrics [36]. For Precision, Recall, and F1 score, detections with confidence scores higher than 0.5 were retained, and the confidence threshold was fixed across all models rather than being tuned separately for each method. The definitions of each evaluation metric are as follows [37]:

(16) $$\mathrm{Precision}=\frac{TP}{TP+FP},\;\mathrm{Recall}=\frac{TP}{TP+FN}$$

(17) $$F1=\frac{2\times \mathrm{Precision}\times \mathrm{Recall}}{\mathrm{Precision}+\mathrm{Recall}}$$

In this context, AP represents the area under the Precision-Recall curve, whereas mAP corresponds to the average of AP across all categories, given by:

(18) $$\mathrm{mAP}=\frac{1}{N}\sum_{c=1}^{N}{\mathrm{AP}}_{c}$$

In the above equations, TP, FP, and FN represent true positives, false positives, and false negatives, respectively, while *N* indicates the total category count.

### 4.3. Ablation Study

A set of ablation studies were conducted on the RH-25 dataset to systematically evaluate the influence of each proposed module on the detection performance of the baseline network. Here, the baseline denotes a CenterNet-based bimodal detector with a single-stack Hourglass backbone and a standard three-branch detection head, using the same fused-image and infrared-image inputs but without the FFA module, HGDH module, or two-stack Hourglass design. First, each module was individually incorporated into the baseline model to analyze its independent contribution. Afterwards, all modules were jointly incorporated to assess the overall performance gain of the proposed method. The comprehensive experimental outcomes are detailed below.

#### 4.3.1. FFA Module

To validate the efficacy of the FFA module, we performed relevant ablation experiments on the RH-25 dataset, with the outcomes summarized in Table 1. With the FFA module integrated into both the input fusion stage and the Joint module, our model yielded a 2.05% gain in mAP@0.5 relative to the baseline on the RH-25 dataset, alongside a 1.2 increase in FPS and an F1 score of 0.87. These results indicate that the FFA module enables effective collaborative representation of different modal information through a three-dimensional weight generation and feature fusion mechanism. Consequently, the model leverages both thermal target responses from infrared images and detailed information from fused images, which mitigates potential information loss arising from direct fusion. In addition, the FFA module further optimizes the feature selection and weighting process, allowing the network to focus more effectively on target-related regions and thus improving the overall detection performance.

The added two-stack Hourglass-only setting in Table 1 is used to separate the effect of backbone capacity from the proposed FFA and HGDH modules. Compared with the baseline, the two-stack Hourglass-only setting brings a limited improvement in mAP@0.5, while the complete model achieves further performance gains after introducing FFA and HGDH. This indicates that the improvement of the complete model is not solely caused by increasing the backbone depth.

**Figure 7:**
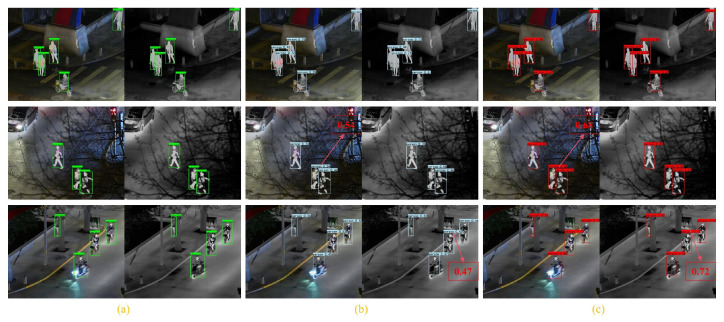
Qualitative comparison of ablation-related detection results in representative low-light and complex-background scenes. (**a**) Ground-truth annotations. (**b**) Detection results of the baseline model. (**c**) Detection results of the proposed model. The highlighted regions show that the proposed model produces stronger target responses and higher confidence scores than the baseline. The individual contributions of FFA and HGDH are quantitatively analyzed in Table 1.

To further support the ablation analysis, qualitative visualization results are provided in Figure 7. The figure compares the ground-truth annotations, the baseline detection results, and the results of the complete proposed model. Since FFA and HGDH mainly operate on intermediate feature representations and heatmap-guided spatial responses, their individual effects are not always directly distinguishable from the final bounding-box visualization. Therefore, the module-level contributions are primarily analyzed through the quantitative ablation results in Table 1. As shown in Figure 7, compared with the baseline, the proposed model maintains similar localization results while producing stronger target responses and higher confidence scores in representative low-light and complex-background scenes.

#### 4.3.2. HGDH Module

Under identical experimental conditions, we performed ablation studies to assess the impact of the HGDH (Heatmap-Guided Detection Head) module on model performance. The results show that, on the RH-25 dataset, introducing HGDH improved the mAP@0.5 of the model by 3.17% over the baseline, while the other evaluation metrics also maintained favorable performance. These results indicate that the HGDH module explicitly selects and enhances target-related features by generating spatial probability masks from the predicted heatmaps, thereby effectively suppressing interference from complex backgrounds. In particular, it improves target localization and recognition in occluded and complex scenes, which further enhances the overall model performance.

According to the ablation results, after embedding the Feature Fusion Attention (FFA) module and the Heatmap-Guided Detection Head (HGDH) into the feature fusion stage and the Joint module of the bimodal baseline model, respectively, the model exhibited gains in both cross-modal feature representation and target-region enhancement. Compared with the baseline model, the improved model achieved a 3.51% increase in mAP@0.5 on the RH-25 dataset, with the F1 score rising to 0.93, while maintaining comparable inference speed under the current experimental setting. These results suggest that the proposed modules contribute to improved cross-modal feature representation and target-region enhancement under low-light and complex-scene conditions.

### 4.4. Comparison Experiments

For the comparative study, we performed extensive evaluations on our custom RH-25 dataset, benchmarking a range of representative models: classical object detectors, state-of-the-art models with reported strong performance from recent years, and the latest bimodal detection architectures. To guarantee fair and comparable outcomes, we conducted all experiments under identical hardware conditions, training epochs, and parameter configurations.

We systematically compared our model against competing approaches on the RH-25 dataset, with detailed outcomes provided in Table 2. To thoroughly assess detection performance across models, we used mAP@0.5 as the primary metric for quantifying detection capability at a 0.5 IoU threshold. Meanwhile, Precision, Recall, and F1 score were also reported to reflect the overall detection performance of each model. In addition, We additionally recorded FPS as a measure of each model’s real-time detection performance. Regarding the selection of comparison models, the one-stage detectors YOLOv8n and YOLOv11n were first included, while Faster R-CNN was chosen as a widely used and stable two-stage detector. In addition, RT-DETR, which is based on a Transformer architecture [38], as well as recent representative bimodal detection models, including FreDFT and IC-Fusion [39], were introduced to improve the comprehensiveness of the comparison experiments.

Based on the comparison results in Table 2, the proposed model achieves the highest mAP@0.5 under the current fixed experimental setting. Compared with lightweight detectors such as YOLOv8n and YOLOv11n, the proposed method obtains higher mAP@0.5, while requiring a larger parameter count and higher computational cost. Compared with Faster R-CNN, the proposed method also achieves a higher mAP@0.5, indicating its effectiveness in low-light and complex-background scenarios. Overall, these results show that the proposed method achieves competitive detection performance on the RH-25 dataset, especially in terms of mAP@0.5. However, its computational efficiency still requires further optimization for practical deployment.

**Figure 8:**
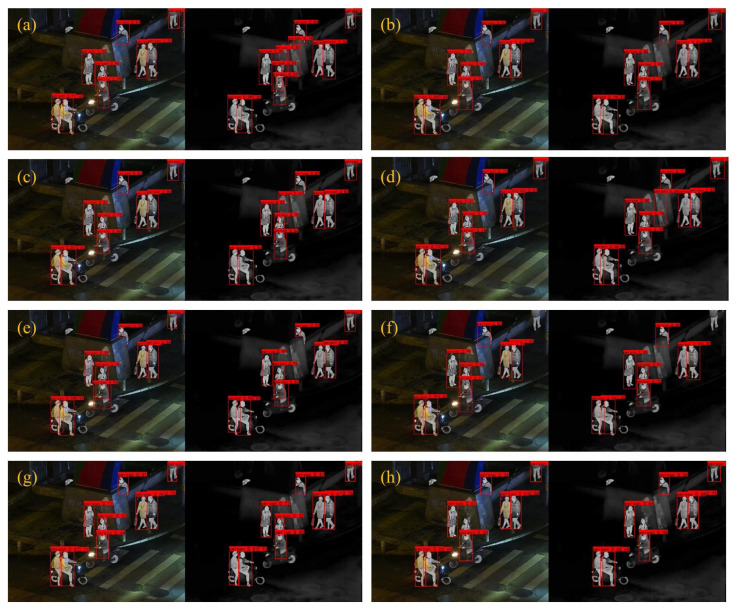
Visualization comparison of detection results from different models on the RH-25 dataset. (**a**) Faster R-CNN; (**b**) RT-DETR; (**c**) YOLOv8n; (**d**) YOLOv11n; (**e**) IC-Fusion; (**f**) FreDFT; (**g**) the proposed model; (**h**) the baseline model.

It is also worth noting that the baseline model shows a large gap between Precision and Recall. This indicates that the baseline tends to make conservative predictions: it produces few false positives and thus achieves high Precision, but it misses some weak-response or partially occluded targets under low-light and complex-background conditions, resulting in relatively low Recall. This can be attributed to the limited representation capability of the single-stack Hourglass backbone and the absence of the proposed FFA and HGDH modules. After introducing the proposed modules and the two-stack Hourglass design, Recall increases from 68.89% to 87.05%, while Precision remains high, indicating that the proposed method improves detection completeness without introducing excessive false positives. Furthermore, Figure 8 offers a visual comparison of detection results across models, illustrating their detection performance under poor illumination and complex environmental scenarios.

**Figure 9:**
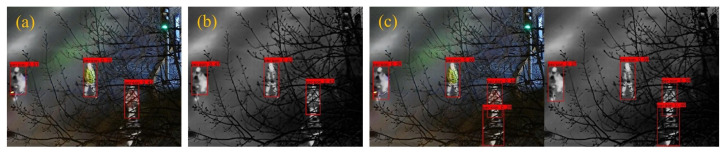
Visualization comparison of detection results under different input modalities on the RH-25 dataset. (**a**) Fused-image input; (**b**) infrared-image input; (**c**) fused-image and infrared-image pair input.

To further examine the effect of dual-modal input on model performance, we performed comparative experiments on the baseline model with fused images, infrared images, and joint inputs. The experimental results are shown in Table 3. It can be observed that, compared to single-modal input, the joint use of both modalities leads to improvements in several evaluation metrics. This indicates that the combination of infrared and fused images provides complementary information for detection, thereby improving detection performance under low-light and complex-background conditions. Additionally, the corresponding visual comparison results are presented in Figure 9.

In summary, the proposed method achieves competitive detection performance on the RH-25 dataset under the current fixed experimental setting. The results suggest that the fused-image and infrared-image inputs, together with the proposed feature fusion and target-region enhancement modules, contribute to improved detection performance compared with the baseline. However, the proposed model still has a relatively large computational cost, and its efficiency requires further optimization for practical deployment and real-time applications.

### 4.5. Generalization Experiments

To further assess the generalization capability of the proposed method, supplementary experiments were conducted on the MFAD dataset. Comprising 12,194 image pairs, this dataset spans six categories: Car, Bus, Truck, Pedestrian, EbikeRider, and Cyclist. The diversity of the MFAD dataset, including various application scenarios and target categories, makes it ideal for evaluating the model’s adaptability across different scenes and multi-class conditions.

The experiments were conducted with the same training strategy and parameter configurations as the main experiments. To guarantee a fair comparison, we performed all experiments under identical hardware and software conditions. In addition to the baseline, two representative comparison detectors, YOLOv11n and IC-Fusion, were further included to provide a more comprehensive evaluation of cross-dataset generalization. As reported in Table 4, the proposed model achieves an mAP@0.5 of 73.12% on the MFAD dataset under the current fixed experimental setting. This result is higher than those of the baseline, YOLOv11n, and IC-Fusion in terms of mAP@0.5, indicating that the proposed method maintains favorable detection performance on a different dataset. These results provide supplementary evidence for the cross-dataset adaptability of the proposed framework under varying data distributions and scenarios.

**Figure 10:**
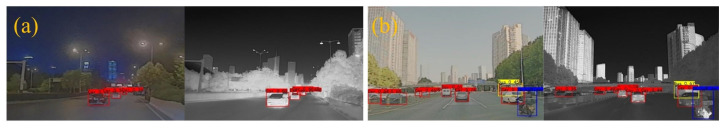
Visualization of the generalization results of the proposed model on the MFAD dataset. (**a**) Detection result under nighttime conditions; (**b**) detection result for multiple target categories under daytime conditions. Red boxes indicate Car, yellow boxes indicate Bus, and blue boxes indicate EbikeRider.

Further qualitative results are shown in Figure 10. The visualization results indicate that the proposed model can detect representative targets under different MFAD scenes, including nighttime and multi-class daytime conditions. These examples provide qualitative support for the cross-dataset evaluation, while the quantitative results in Table 4 remain the main basis for comparison.

## 5. Conclusions

This study proposes an improved CenterNet-based multimodal object detection method for low-light and complex-background scenes. The proposed method constructs a dual-source input using fused images and infrared images, introduces infrared Haar wavelet structural-detail priors through the FFA module, and employs the HGDH module to enhance target-related responses in the detection stage. In addition, a two-stack Hourglass backbone is adopted for multi-scale feature modeling. Experimental results on the RH-25 dataset show that the proposed method achieves competitive detection performance under the fixed experimental setting, while the supplementary MFAD experiments indicate its potential cross-dataset adaptability. Future work will focus on lightweight structural design, inference speed optimization, and deployment in practical scenarios.

## Figures and Tables

**Table 1:** Ablation study results of each module on the RH-25 dataset.

Baseline	Hourglass (Two-Stack)	FFA	HGDH	Precision (%)	Recall (%)	mAP@0.5 (%)	F1	FPS
✓				99.44	68.89	93.12	0.81	12.11
✓	✓			99.27	74.34	93.89	0.85	12.76
✓		✓		99.07	76.80	95.17	0.87	13.31
✓			✓	**99.82**	79.12	96.29	0.88	13.75
✓	✓	✓	✓	98.42	**87.05**	**96.63**	**0.93**	**15.24**

Note: ✓ indicates that the corresponding module or design is used. Bold values indicate the best results in each metric.

**Table 2:** Quantitative comparison of different detection models on the RH-25 dataset.

Model	Modality	Precision (%)	Recall (%)	mAP@0.5 (%)	F1	Params (M)	FLOPs (G)	FPS
YOLOv8n [40]	Fused + IR	95.19	89.24	88.28	0.92	3.01	4.10	25.20
YOLOv11n [41]	Fused + IR	95.46	90.08	89.02	0.93	2.59	3.22	20.14
Faster R-CNN [20]	Fused + IR	68.39	**95.42**	94.64	0.80	28.28	257.75	21.12
RT-DETR [24]	Fused + IR	88.11	93.52	91.38	0.91	32.81	54.00	**28.43**
FreDFT [42]	Fused + IR	56.78	91.67	89.16	0.70	23.52	36.72	22.47
IC-Fusion [39]	Fused + IR	55.36	92.03	89.93	0.69	26.24	113.62	15.12
Baseline [15]	Fused + IR	**99.44**	68.89	93.12	0.81	95.43	251.43	12.11
Ours	Fused + IR	98.42	87.05	**96.63**	**0.93**	192.50	528.28	15.24

Note: Bold values indicate the best results in each metric.

**Table 3:** Ablation study results of different input modalities on the RH-25 dataset.

Modality	Precision (%)	Recall (%)	mAP@0.5 (%)	F1	FPS
Fused	99.17	69.32	91.78	0.82	12.03
IR	99.05	**72.55**	91.65	**0.84**	**12.14**
Fused + IR	**99.44**	68.89	**93.12**	0.81	12.11

Note: Bold values indicate the best results in each metric.

**Table 4:** Generalization performance comparison on the MFAD dataset.

Model	Modality	Precision (%)	Recall (%)	mAP@0.5 (%)	F1
YOLOv11n [41]	Fused + IR	**90.31**	**71.14**	71.69	**0.80**
IC-Fusion [39]	Fused + IR	77.58	68.43	70.74	0.73
Baseline [15]	Fused + IR	89.59	56.63	70.90	0.69
Ours	Fused + IR	90.15	70.46	**73.12**	0.79

Note: Bold values indicate the best results in each metric.

## Data Availability

The list of original LLVIP image filenames corresponding to the selected RH-25 samples and the supplementary Python (v3.8) script for RH-25 subset construction are provided as Supplementary Material to support reproducibility.

## Supplementary Materials

The following supporting information can be downloaded at: https://www.mdpi.com/article/10.3390/s26123735/s1, Supplementary File S1: Python script for RH-25 sample selection from the public LLVIP dataset and the corresponding original LLVIP image filename list.

## Abbreviations

The following abbreviations are used in this manuscript:

CenterNet	Objects-as-Points Detector
FFA	Feature Fusion Attention
HGDH	Heatmap-Guided Detection Head
